# Steatosis indices and penile vascular health: a metabolic association linking liver and vascular phenotypes

**DOI:** 10.1007/s40618-026-02889-1

**Published:** 2026-04-10

**Authors:** Andrea Delbarba, Massimiliano Ugoccioni, Elisa Gatta, Giorgio Tiecco, Eugenia Quiros-Roldan, Andrea Graziani, Ilenia Pirola, Alberto Ferlin, Carlo Cappelli

**Affiliations:** 1 Department of Clinical and Experimental Sciences, SSD Endocrinologia, University of Brescia, ASST Spedali Civili Brescia, Piazzale Spedali Civili n°1, Brescia, 25121 Italy; 2 Department of Internal Medicine and Therapeutics, University of Pavia, Pavia, Italy; 3 Department of Clinical and Experimental Sciences, SD of Infectious and Tropical Diseases, University of Brescia and ASST Spedali Civili di Brescia, Brescia, Italy; 4 Unit of Andrology and Reproductive Medicine, Department of Medicine, University of Padova, Padova, Italy

**Keywords:** Hepatic steatosis, Vascular erectile dysfunction, FLI, HSI, DSI

## Abstract

**Objective:**

To examine the association between non-invasive steatosis indices—Fatty Liver Index (FLI), Hepatic Steatosis Index (HSI), and Dallas Steatosis Index (DSI)—and Penile Color Doppler Ultrasonography (PCDU) derived parameters of vasculogenic erectile dysfunction, including peak systolic velocity (PSV) and intima–media thickness (IMT).

**Methods:**

In this retrospective study, 96 men evaluated for erectile dysfunction (ED) underwent dynamic PCDU after intracavernosal alprostadil injection. Clinical, biochemical, and metabolic data were collected. Associations between steatosis indices and PCDU parameters were analysed. Multiple regression identified independent predictors of PSV, and ROC analysis assessed the performance of FLI in detecting reduced PSV (< 35 cm/s).

**Results:**

Reduced PSV was observed in 24% of participants and was associated with older age, higher FSH and LH levels, lower total and free testosterone, and higher FLI values, while HSI and DSI did not differ between groups. FLI correlated inversely with PSV (*R* = − 0.305; *p* = 0.003). In multivariable analysis, only FLI (*p* = 0.031) independently predicted PSV. ROC curve analysis indicated that an FLI threshold ≥ 67.6 identified reduced PSV with 56% sensitivity and 82% specificity.

**Conclusions:**

FLI is independently associated with impaired penile arterial inflow and may represent a practical, non-invasive marker of vascular andrological risk. These findings suggest that FLI could assist early risk stratification in men with ED.

**Supplementary Information:**

The online version contains supplementary material available at 10.1007/s40618-026-02889-1.

## Introduction

Erectile dysfunction (ED) is defined as the persistent or recurrent inability to achieve or maintain an erection sufficient for satisfactory sexual performance [1]. The most recent epidemiological studies show that ED affects approximately 24% of adult men in the United States, with a prevalence increasing proportionally with age, reaching up to 52% after 75 years old [2]. Similar age-related trends are observed internationally, with prevalence rates exceeding 40% in men over 60 and approaching 80% in men over 70 [3]. ED is strongly associated with cardiovascular risk factors such as diabetes, hypertension, obesity, and smoking [4]. As widely recognized, vasculogenic erectile dysfunction (VED)—defined as a subtype of organic ED caused by impaired penile vascular function due to arterial insufficiency or veno-occlusive dysfunction—represents the most prevalent cause of ED [5].

According to the 2023 Guidelines of the Italian Society of Andrology and Sexual Medicine (SIAMS), dynamic penile color Doppler ultrasonography (PCDU) after intracavernosal vasoactive injection represents the test of choice to measure penile blood flow, assess penile vascular function, and detect structural abnormalities. In particular, PCDU is recommended in men with ED when a vascular contribution is suspected, especially in the presence of cardiovascular or metabolic risk factors, inadequate response to first-line therapy, early-onset ED, or prior to second-line or invasive treatments [6].

Recent studies suggest that a reduction in cavernous artery peak systolic velocity (PSV) and/or the increase of and intima–media thickness (IMT) following Prostaglandin E₁ (PGE₁) administration may represent a marker of systemic vascular damage and an early indicator of major cardiovascular events [7, 8]. In accordance, the *Princeton IV Consensus Recommendations for the Management of Erectile Dysfunction and Cardiovascular Disease* identified erectile dysfunction as an independent risk factor for cardiovascular disease and recommended cardiovascular risk screening in men with VED [9].

It is well known that also hepatic steatosis is strongly correlated to a heightened risk of cardiovascular disease [10], and in recent years, various non-invasive indices have been proposed to estimate the presence and severity of hepatic steatosis. Among them, the Fatty Liver Index (FLI), Hepatic Steatosis Index (HSI), and Dallas Steatosis Index (DSI) are validated indices used for large-scale screening studies [11].

Recent evidence highlights the interplay between hepatic steatosis indices and vascular health; HSI and FLI have been demonstrated to be independently associated with increased carotid intima–media thickness and higher prevalence of carotid plaque [12]. Similarly, FLI seems to predict cardiovascular events, particularly in individuals under 50 years [13]. Elevated HSI is associated to metabolic dysfunction driven by systemic inflammation, insulin resistance, and endothelial impairment—key mechanisms underlying VED [14, 15]. To date, the few studies exploring the relationship between erectile dysfunction and steatosis-related indices rely exclusively on questionnaire-based assessments—such as the International Index of Erectile Function-5 (IIEF-5) —rather than on instrumental data, suggesting a potential association between FLI and ED severity [16, 17].

PSV provides a direct, objective, and reproducible parameter for identifying VED, while excluding non-organic causes and overcoming the inherent subjectivity of questionnaire-based assessments. To the best of our knowledge, no study to date has investigated the association between steatosis indices and objective instrumental assessed by PCDU. In light of this, our study aims to investigate the association among FLI, HSI and DSI and PSV and IMT in the evaluation of ED, in order to assess its potential use as a non-invasive marker of vascular andrological risk.

## Methods

### Patients

This single-centre retrospective observational study included male patients aged ≥ 18 years referred to our outpatient clinic for the evaluation of ED, whose electronic medical records documented PDCU performed between November 1, 2022, and January 31, 2025, and gave their written informed consent to participate in the study. In accordance with routine clinical practice, PCDU was performed in patients with an IIEF-5 score ≥ 12, in whom a vascular aetiology of ED was clinically suspected and instrumental assessment was considered appropriate.

Patients with a history of alcoholism and/or ethanol consumption greater than 20 g/day, as well as those with liver cirrhosis or other chronic liver diseases (including viral hepatitis, autoimmune hepatitis, primary sclerosing cholangitis, primary biliary cholangitis, drug-induced liver disease, hemochromatosis, Wilson’s disease, or alpha-1 antitrypsin deficiency) were excluded. Additional exclusion criteria included a history of pelvic surgery or trauma, the use of medications known to interfere with erectile function (i.e. non-vasodilating B-blockers, thiazide diuretics, antidepressants, antipsychotics, anti-androgenic agents), liver metabolism or to promote hepatic steatosis (i.e. amiodarone, methotrexate, valproate, chronic corticosteroids), hypogonadism in any form (including patients receiving testosterone replacement therapy), and incomplete clinical or instrumental data.

The primary outcome was to assess the association between PCDU-derived parameters of penile vascular function (PSV and IMT) and non-invasive hepatic steatosis indices, including FLI, HSI, and DSI.

The study was approved by the local Ethics Committee (n. 5184).

### Clinical evaluation

During the clinical evaluation, detailed family and medical histories were obtained, and a comprehensive physical examination including weight, height, and waist circumference was performed and recorded in the electronic medical chart. Body mass index (BMI) was calculated. All patients also completed the IIEF-5 questionnaire. Score for the IIEF-5 ranges from 5 to 25, and we classified ED into five categories based on the following ranges: severe (5–7), moderate (8–11), mild to moderate (12–16), mild (17–21), and no ED (22–25) [18].

### Dynamic penile color Doppler duplex ultrasound

All patients underwent PCDU following an intracavernous injection of 10 µg alprostadil [19]. All diagnostic procedures were performed by an experienced physician with a case load of more than 100 procedures per year. The procedure was performed using a Samsung H60 ultrasound system, equipped with a high-resolution linear array transducer with a frequency range of 7.5–14 MHz. Both PSV and IMT were assessed bilaterally 20 min after PGE_1_ injection. PSV was calculated as the average of the two values recorded at 20 min, and a threshold of < 35 cm/s was used to define penile cavernosal arterial insufficiency [19]. The presence of cavernous artery plaques was defined as a thickness ≥ 0.3 mm on at least one side [20].

### Biochemical analyses

All blood samples were collected in the morning after an overnight fast. Total testosterone (T; normal range: 2.49–8.36 mcg/L; limit of quantitation 0.12 ng/mL), luteinizing hormone (LH; normal range: 1.7–8.6 IU/L; limit of quantitation 1 mIU/mL) and follicle-stimulating hormone (FSH; normal range: 1.5–12.4 IU/L; limit of quantitation 1 mIU/mL), were assessed using standardized assays. Circulating free testosterone (cFT) was calculated based on total testosterone, sex hormone-binding globulin (SHBG; normal range: 18.8–54.1 nmol/L; limit of detection 0.35 nmol/L), and albumin (normal range: 3.1–5.2 g/dL) levels according to the Vermeulen formula [21]. These hormonal assays were conducted using chemiluminescence-based techniques (Roche Diagnostics, Cobas^®^ E411 and E801 analysers). The intra- and inter-assay coefficients of variation for T, FSH, LH, and SHBG were below 5%. Patients were defined as hypogonadal if they were already on testosterone replacement therapy or if baseline tests showed total testosterone levels < 3.5 ng/mL and/or free testosterone levels < 63 pg/mL [22, 23].

Biochemical markers of metabolism were also measured, including fasting plasma glucose (normal range: <100 mg/dL), aspartate aminotransferase (GOT; normal value 18–34 U/L), alanine aminotransferase (GPT; normal value: 10–50 U/L), gamma-glutamyl transferase (GGT; normal value: 10–71 U/L), total cholesterol (≤ 190 mg/dL), high-density lipoprotein cholesterol (HDL; normal value ≥ 40 mg/dL), and triglycerides (≤ 150 mg/dL). Low-density lipoprotein cholesterol (LDL) was calculated according to Friedwald’s formula [24].

All analyses were performed in the same Laboratory.

### Assessment of hepatic steatosis

Non-invasive hepatic steatosis indices were calculated according to the following formula:

- FLI [25] = (e^0.953 × loge(triglycerides) + 0.139 × BMI +^ ^0.718 × loge(GGT) + 0.053 × waist circumference −15.745^)/(1 + ^e0.953 × loge(triglycerides)^ ^+0.139 × BMI + 0.718 × loge(GGT)^ ^+ 0.053 × waist circumference −15.745^) × 100.
- HIS [26] = 8 × ALT/AST ratio + BMI + 2 (if T2DM) + 2 (if female).
- DSI [27] = − 9.4 + 0.316 (if age ≥ 50 and female) + 2.4 (if known diabetes) + 0.02 * (equals 0 if diabetic; if not diabetic equals the glucose concentration in mg/dL) + 0.3 (if known hypertension) + 0.5 (if Hispanic/Asian/Other race/ethnicity) + Ln triglycerides in mg/dL + 0.4 if ALT 13.5-19.49 IU/L + 1.1 if ALT 19.5–40 IU/L + 1.5 if ALT > 40 IU/L + 0.7 if not Black and BMI 25–27.49 kg/m^2^ and + 1.4 if not Black and BMI 27.5–34.9 kg/m^2^ + 1.9 if not Black and BMI 35–37.49 kg/m^2^ + 2.6 if not Black and BMI > 37.5 kg/m^2^ − 0.2 if Black and BMI 25–27.49 kg/m^2^ +0.8 if Black and BMI 27.5–34.9 kg/m^2^ + 0.8 if Black and BMI 35–37.49 kg/m^2^ + 1.8 if Black and BMI > 37.5 kg/m^2^.

### Statistical analyses

Data are presented as mean ± standard deviation for normally distributed variables, as median (interquartile range) for non-normally distributed variables, and as categorical when appropriate. Normality was assessed using the Shapiro-Wilk test. Continuous variables were compared using Student’s t-test or the Mann-Whitney U test, as appropriate. Categorical variables were compared using the chi-square test or Fisher’s exact test, as applicable.

Correlations between PCDU-derived parameters (PSV and IMT) and clinical, biochemical, and metabolic variables was first explored using correlation analysis, with the choice of Pearson’s or Spearman’s rank correlation coefficient according to data distribution. A multiple linear regression analysis was then performed with PSV as the dependent variable. Covariates were selected based on their significant univariate associations with PSV and the absence of overlap with the components used to calculate the FLI, in order to adjust for potential confounding while avoiding overadjustment. Sensitivity analyses were additionally performed by including BMI and waist circumference separately in the multivariable model, given their potential confounding role despite being components of the FLI formula.

Receiver operating characteristic (ROC) curve analysis was performed to evaluate the discriminative ability of FLI with respect to pathological PSV, and the optimal cut-off point was identified using Youden’s index (sensitivity + specificity − 1). The area under the curve (AUC) was calculated with 95% confidence intervals. All statistical analyses were performed using SPSS 20.0 software (SPSS, Inc., Evanston, IL, USA) and R Studio 4.2.2. A significance level of *p* ≤ 0.05 was set.

The results are reported in compliance with the STROBE reporting guidelines for cross-sectional studies.

## Results

A total of 96 patients (mean age 51.7 ± 11.1 years) were enrolled. All participants exhibited impaired IIEF-5 scores [median 13 (IQR 8–17)]; specifically, 27 patients (28%) had severe ED, 13 (13%) moderate ED, 29 (31%) mild-to-moderate ED, and 27 (28%) mild ED. Clinical and biochemical characteristics are summarized in Table 1. Briefly, 28 participants (29%) had well-controlled hypertension, 19 (20%) were receiving lipid-lowering therapy, 13 (14%) were on hypoglycaemic agents, and 14 (15%) were classified as obese. Active smoking was reported in 35 subjects (36%).

**Table 1:** Clinical characteristics of the patients

	All patients (*n* = 96)	Normal PSV (*n* = 73)	Reduced PSV (*n* = 23)	*p*	Normal IMT (*n* = 19)	Increased IMT (*n* = 67)	*p*
**Age at study entry (years)**	51.7 ± 11.1	49.2 ± 10.5	60.6 ± 7.8	< 0.001	44.1 ± 12.1	53.5 ± 9.9	0.022
**No. of smokers**	35 (36%)	24 (33%)	11 (48%)	0.194	8 (42%)	25 (37%)	0.144
**WC (cm)**	96.6 ± 13.2	94.7 ± 12.9	103.0 ± 12.7	0.166	94.0 ± 12.8	95.5 ± 12.4	0.287
**BMI (Kg/m** ^**2**^ **)**	26.2 (24.3 to 28.0)	25.5 (23.8 to 28.0)	26.9 (25.9 to 33.2)	0.531	25.7 (24.3 to 28.0)	26.1 (23.8 to 27.9)	0.429
**Systolic pressure**	140 (126 to 149)	140 (125 to 148)	140 (127 to 167)	0.375	140 (123 to 148)	138 (126 to 150)	0.979
**Diastolic pressure**	96 (89 to 103)	85 (77 to 90)	90 (80 to 103)	0.103	84 (77 to 91)	85 (79 to 91)	0.931
**FSH (IU/L)**	5.6 (3.7 to 10.2)	4.9 (3.3 to 8.1)	10.2 (5.4 to 22.9)	< 0.001	3.8 (3.4 to 4.5)	6.6 (4.0 to 10.3)	0.040
**LH (IU/L)**	7.2 (5.1 to 8.3)	6.9 (4.8 to 7.8)	9.0 (6.1 to 17.5)	0.004	5.9 (5.3 to 6.9)	7.4 (5.0 to 8.4)	0.347
**T (mcg/L)**	4.7 (4.2 to 5.8)	4.9 (4.3 to 6.1)	4.5 (3.8 to 4.7)	0.003	4.7(4.4 to 4.9)	5.2 (4.5 to 6.2)	0.123
**cFT (ng/L)**	90.4 ± 25.1	95.2 ± 22.8	73.8 ± 26.9	0.004	84.0 (74.6 to 105.0)	94.5 (79.0 to 110.0)	0.572
**SHBG**	41.0 (32.0 to 53.0)	40.5 (32.0 to 53.3)	54.4 (46.0 to 75.0)	0.759	39.0 (35.0 to 47.5)	44.5 (33.5 to 54.5)	0.230
**Glycemia (mg/dL)**	97.0 (90.0 to 109.0)	95.0 (88.7 to 106.3)	109.0 (922.0 to 111.5)	0.043	93.0 (88.0 to 100.0)	98.5 (90.0 to 109.0)	0.252
**Total cholesterol (mg/dL)**	187.0 ± 33.8	186.5 ± 33.2	188.8 ± 37.5	0.395	198.0 ± 35.6	183.3 ± 33.6	0.247
**HDL (mg/dl)**	47.0 (39.0 to 59.0)	50.5 (39.0 to 60.0)	42.0 (39.5 to 52.0)	0.180	51.0 (44.5 to 53.0)	45.0 (39.0 to 60.0)	0.458
**LDL (mg/dl)**	116.5 ± 32.6	116.6 ± 32.8	116.1 ± 32.9	0.315	128.0 (120.0 to 136.0)	108.7 (88.5 to 142.4)	0.711
**Triglycerides (mg/dL)**	87.0 (66.0 to 126.0)	78.5 (65.0 to 116.3)	126.0 (78.5 to 167.5)	0.064	76.0 (67.5 to 92.5)	82.5 (65.0 to 125.0)	0.320
**GOT (U/L)**	25.0 (17.0 to 38.0)	24.0 (20.7 to 30.0)	26.0 (23.0 to 37.5)	0.600	27.0 (22.5 to 30.0)	24.0 (21.5 to 31.0)	0.508
**GPT (U/L)**	25.0 (22.0 to 32.0)	25.5 (22.0 to 32.3)	21.0 (17.5 to 54.5)	0.843	24.0 (23.0 to 29.5)	26.0 (20.5 to 37.0)	0.485
**GGT (U/L)**	25.0 (21.0 to 38.0)	25.0 (16.5 to 37.0)	33.0 (22.5 to 50.5)	0.193	21.0 (12.0 to 34.5)	25.5 (18.0 to 42.5)	0.198
**Obesity**	14 (15%)	10 (14%)	4 (17%)	0.737	1 (5%)	10 (14%)	1.239
**Arterial hypertension**	28 (29%)	15 (21%)	13 (56.5%)	< 0.001	4 (21%)	21 (31%)	0.285
**Dyslipidaemia**	19 (20%)	11 (15%)	8 (34%)	0.039	5 (26%)	13 (19%)	0.427
**Diabetes mellitus**	13 (14%)	10 (14%)	3 (13%)	0.999	2 (10%)	11 (16%)	0.413
**FLI**	43.1 (20.8 to 67.4)	35.0 (16.0 to 57.0)	68.8 (37.0 to 86.5)	0.047	33.0 (15.0 to 51.0)	36.0 (22.0 to 66.0)	0.117
**HSI**	34.8 (32.4 to 38.2)	35.0 (32.0 to 37.0)	34.3 (32.6 to 45.4)	0.921	34.6 (33.0 to 36.0)	33.7 (32.1 to 38.9)	0.087
**DSI**	-1.1 (-1.9 to -0.5)	-1.4 (-2.0 to -0.6)	-0.7 (-1.3 to 0.4)	0.134	-1.5 (-1.8 to -1.0)	-1.2 (-1.9 to -0.5)	0.186
**IIEF-5**	13.0 (7.5 to 17.0)	14.0 (9.8 to 17.2)	7.0 (5.0 to 13.5)	0.162	15.0 (12.0 to 17.5)	13.0 (7.5 to 17.5)	0.190
**PSV**	42.0 (37.0 to 55.0)	45.5(40.0 to 57.0)	30.0 (28.0 to 32.0)	< 0.001	48.0 (41.0 to 58.0)	42.5 (38.0 to 55.0)	0.143

PSV: peak systolic velocity; IMT: intima-media thickness; WC: waist circumference; BMI: body mass index; FSH: follicle-stimulating hormone; LH: luteinizing hormone; T: testosterone; cFT: circulating free testosterone; SHBG: sex hormone binding globulin; HDL: high-density lipoprotein cholesterol; LDL: low-density lipoprotein cholesterol; GOT: aspartate aminotransferase; GPT: alanine aminotransferase; GGT: gamma glutamyl transferase; FLI: fatty liver index; HSI: Hepatic Steatosis Index; DSI: Dallas Steatosis Index; IIEF-5: International Index of Erectile Function-5

Data are expressed as mean value ± SD, median [Q1–Q3] or absolute number (percentage)

Overall, 23/96 patients (24%) showed reduced PSV. These individuals were significantly older than those with normal PSV (60.6 ± 7.8 vs. 49.2 ± 10.5 years; *p* < 0.001), exhibited higher FSH [10.2 (5.4–22.9) vs. 4.9 (3.3–8.1) IU/L; *p* < 0.001] and LH levels (11.6 ± 7.2 vs. 6.5 ± 2.2 IU/L; *p* = 0.004), and had lower TT [4.5 (3.8–4.7) vs. 4.9 (4.3–6.1) mcg/L; *p* = 0.003] and cFT concentrations (73.8 ± 26.9 vs. 95.2 ± 22.8 mcg/L; *p* = 0.004). Arterial hypertension and dyslipidaemia were significantly more prevalent in individuals with reduced PSV compared with those with normal PSV (hypertension: 56.5% vs. 21.0%, *p* < 0.001; dyslipidaemia: 34.0% vs. 20.0%, *p* = 0.039; Fisher’s exact test). Patients with reduced PSV displayed significantly higher FLI values [68.8 (37–86.5) vs. 35 (16–57); *p* = 0.047]. Conversely, DSI [–0.65 (–1.3 to 0.4) vs. − 1.4 (–2.0 to − 0.62); *p* = 0.134] and HSI [34.3 (32.6–45.4) vs. 35 (32–37); *p* = 0.921] were comparable between groups.

The prevalence of elevated transaminase levels was numerically higher among patients with reduced PSV compared with those with normal PSV (35.0% vs. 17.8%), although this difference did not reach statistical significance (*p* = 0.097).

IMT data were available for 86 subjects, of whom 67 (77%) showed increased IMT. Patients with pathological IMT were significantly older than those with normal IMT (53.5 ± 9.9 vs. 44.1 ± 12.1 years; *p* = 0.022) and had higher FSH levels [6.6 (4–10.3) vs. 3.8 (3.4–4.5) IU/L; *p* = 0.040]. However, in a multivariable model including both age and FSH, neither variable was independently associated with IMT (age: F = 0.024, *p* = 0.880; FSH: F = 1.096, *p* = 0.429). All other parameters were similar between groups. FLI [36 (22–66) vs. 33 (15–51); *p* = 0.117], DSI [–1.2 (–1.9 to − 0.5) vs. − 1.5 (–1.8 to − 1); *p* = 0.186], and HSI [33.7 (32.1–38.9) vs. 34.6 (33–36); *p* = 0.087] were also superimposable (Table 1).

**Fig. 1:**
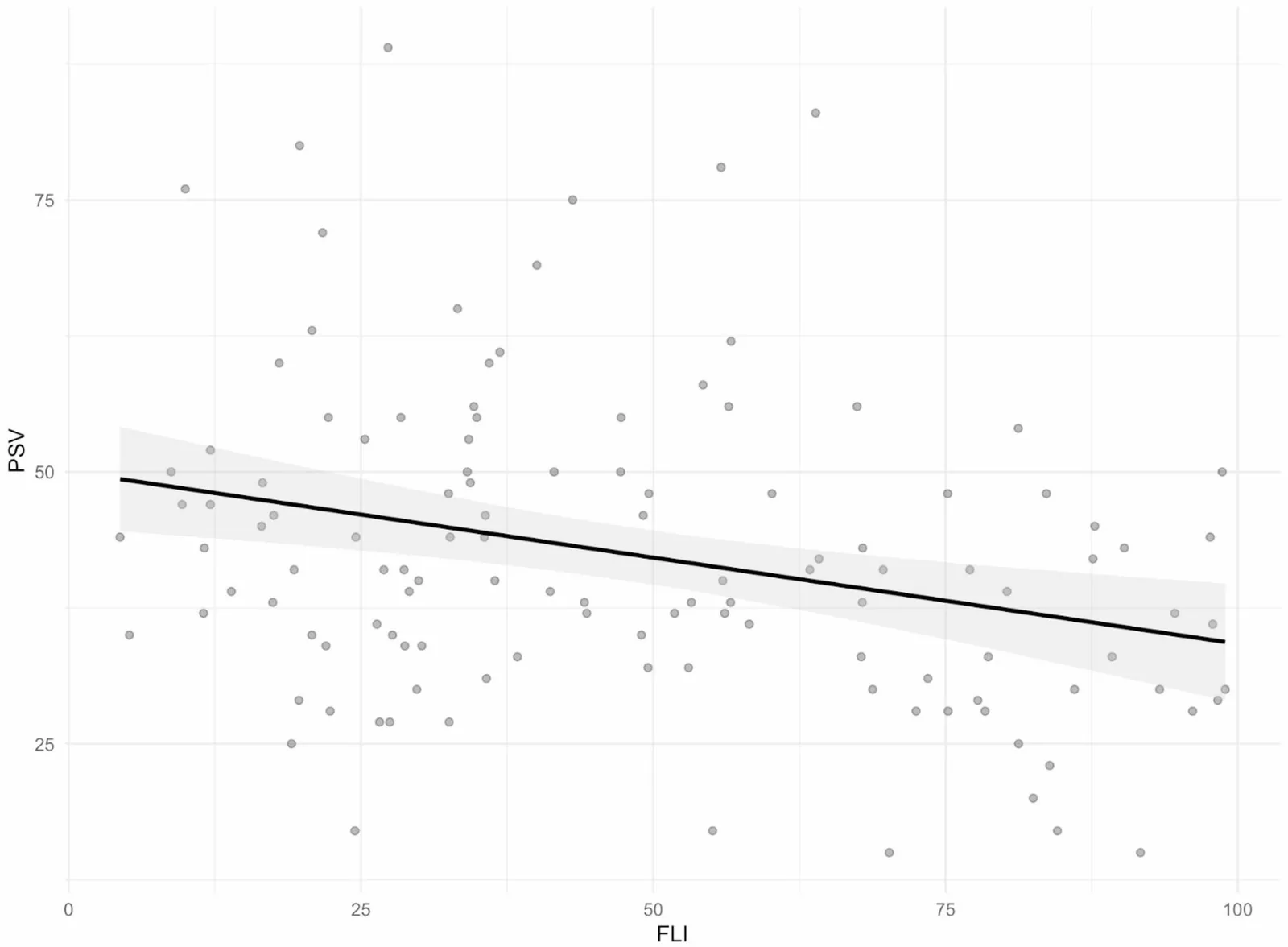
Scatterplot showing the univariable linear regression between fatty liver index (FLI) and peak systolic velocity (PSV). The black line represents the fitted regression line, and the shaded area indicates the 95% confidence interval of the estimate

Correlations between PCDU parameters and clinical and biochemical variables are reported in Table 2. Briefly, age (ρ = − 0.328; *p* = 0.001), waist circumference (ρ = − 0.276; *p* = 0.007), GPT (ρ = − 0.208; *p* = 0.042), GGT (ρ = − 0.224; *p* = 0.028), LH levels (ρ = − 0.292; *p* = 0.005) and diastolic blood pressure (ρ = -0.250; *p* = 0.016) were all negatively associated with PSV. Conversely, cFT (ρ = 0.288; *p* = 0.010) and TT (ρ = 0.208; *p* = 0.044) showed positive correlations. IMT correlated positively with age (ρ = 0.262; *p* = 0.016) and SHBG (ρ = 0.285; *p* = 0.016). Finally, FLI was inversely correlated with PSV (ρ = − 0.279; *p* = 0.007) (Fig. 1), whereas neither HSI nor DSI showed significant associations.

**Table 2:** Correlation matrix between peak systolic velocity and intima–media thickness and biochemical variables

	ρ PSV	*p*	ρ IMT	*p*
HSI	-0.146	0.161	0.127	0.246
DSI	-0.151	0.149	0.168	0.152
FLI	-0.279	0.007	0.140	0.201
Age	-0.328	0.001	0.262	0.016
BMI	-0.118	0.258	0.049	0.654
WC	-0.276	0.007	0.146	0.183
Glycemia	-0.174	0.096	0.200	0.067
Total Cholesterol	0.124	0.238	-0.023	0.836
Triglycerides	-0.162	0.119	-0.039	0.772
LDL	0.057	0.593	-0.019	0.870
GOT	− 0.012	0.906	0.100	0.363
GPT	-0.082	0.433	0.142	0.194
GGT	-0.224	0.058	0.172	0.115
FSH	-0.160	0.139	0.187	0.100
LH	-0.271	0.010	0.206	0.065
T	0.208	0.044	0.151	0.167
cFT	0.281	0.013	-0.068	0.576
SHBG	0.011	0.920	0.285	0.016
IIEF-5	0.203	0.081	-0.074	0.550
Systolic pressure	-0.096	0.365	0.043	0.701
Diastolic pressure	-0.250	0.016	-0.019	0.863

PSV: peak systolic velocity; IMT: intima-media thickness; HSI: Hepatic Steatosis Index; DSI: Dallas Steatosis Index; FLI: fatty liver index; LDL: low-density lipoprotein cholesterol; GOT: aspartate aminotransferase; GPT: alanine aminotransferase; GGT: gamma glutamyl transferase; FSH: follicle-stimulating hormone; LH: luteinizing hormone; T: testosterone; cFT: calculated free testosterone; SHBG: sex hormone binding globulin; IIEF-5: International Index of Erectile Function-5

In a multiple linear regression model with PSV as the dependent variable and FLI, age, diastolic blood pressure, LH, TT, and cFT as independent variables—adjusted for ED severity based on IIEF-5 categorization—FLI remained the only significant predictor of PSV (B = − 0.287; 95% CI − 0.317 to − 0.016; *p* = 0.031) (Table 3). In sensitivity analyses including BMI or waist circumference separately in the multivariable model, the association between FLI and PSV was attenuated and did not reach statistical significance. In these models, neither BMI nor waist circumference were independently associated with PSV (Supplementary Table S1).

**Table 3:** Linear regression results for associations between PSV and FLI, age, GGT, LH and cFT

Covariate	Unstandardized B	Standardized B	95% CI for standardized B	*p*
FLI	-0.166	-0.287	-0.317 to -0.016	0.031
Age	-0.179	-0.117	-0.569 to 0.212	0.364
Diastolic pressure	-0.061	-0.121	-0.180 to 0.057	0.303
LH	-0.277	-0.088	-1.057 to 0.503	0.481
T	-0.661	-0.068	-3.984 to 2.662	0.693
cFT	0.095	0.149	-0.124 to 0.313	0.389

FLI: fatty liver index; LH: luteinizing hormone; T: total testosterone; cFT: calculated free testosterone

Regression coefficients (B), 95% confidence intervals (CI), and p-values are presented for unadjusted models and models adjusted for potential confounders (ED severity based on IIEF-5 categorization)

**Fig. 2:**
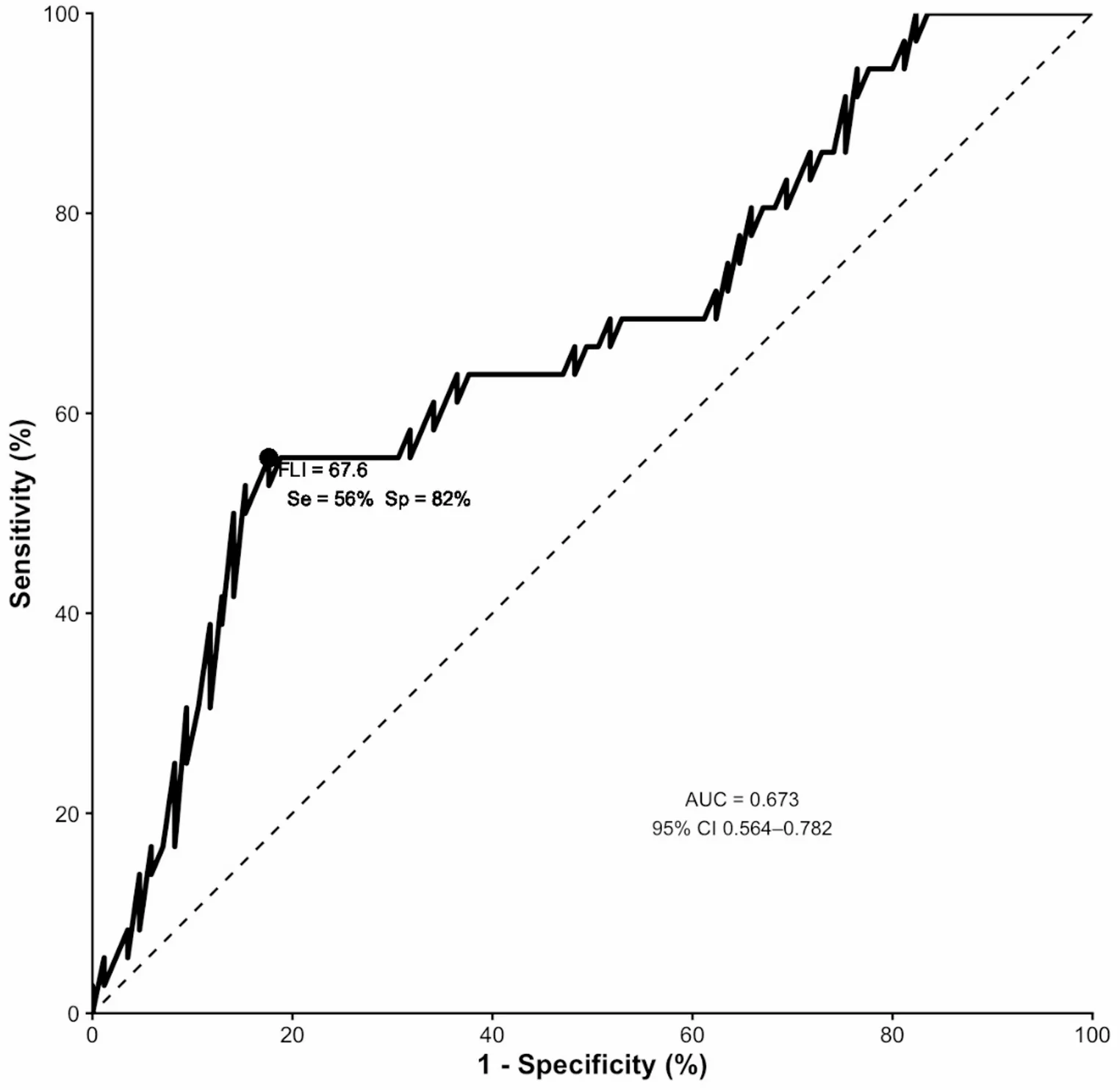
Receiver operating characteristic (ROC) curve of the fatty liver index (FLI) for the identification of pathological peak systolic velocity (PSV < 35 cm/s). The area under the curve (AUC) was 0.673. The black dot indicates the optimal cut-off derived from Youden’s index (FLI = 67.6), corresponding to a sensitivity of 0.56 and a specificity of 0.82

Finally, an exploratory ROC curve analysis indicated that an FLI threshold ≥ 67.6 was able to identify patients with reduced PSV with a sensitivity of 56% (95% CI 39–72) and a specificity of 82% (95% CI 74–91) (Fig. 2).

## Discussion

Our analysis shows that the FLI is significantly associated with reduced PSV after multivariable adjustment, representing, to our knowledge, the first instrument-based observation demonstrating an association between steatosis-related metabolic alterations with objectively measured penile vascular impairment. While liver steatosis in the present study was estimated using surrogate indices based on simple anthropometric and metabolic parameters, such indices were intentionally chosen for their non-invasive nature, reproducibility, and broad use in large-scale and real-world clinical settings, where they serve as practical tools to identify patients at increased metabolic and vascular risk. Importantly, within this framework, the FLI should be interpreted primarily as an integrated marker of global metabolic dysfunction rather than as a liver-specific or mechanistically independent indicator. In this context, the present study provides an objective, instrument-based evaluation of penile vascular function and should be regarded as hypothesis-generating, given its retrospective and cross-sectional design. In our cohort, patients with reduced PSV showed a numerically higher prevalence of elevated transaminase levels; however, this difference did not reach statistical significance, suggesting that inflammatory liver injury is unlikely to be the main driver of the observed association between FLI and penile vascular parameters.

Importantly, no steatosis-related index was associated with cavernous artery IMT. This negative finding underscores a clear distinction between functional and structural vascular outcomes, indicating that steatosis-related metabolic alterations in the present cohort were associated with impaired penile vascular function, as reflected by reduced peak systolic velocity, but not with markers of established arterial remodelling. Accordingly, these results should not be extrapolated to systemic atherosclerotic burden or structural macrovascular disease.

Previous studies have relied exclusively on questionnaire-derived measures of erectile function—primarily the IIEF-5 [16, 17]—and thus were unable to link steatosis indices to objective vascular endpoints. The present study expands the available evidence by integrating steatosis indices with a validated Doppler-based assessment of penile arterial flow [6].

FLI, originally proposed by Bedogni et al. in 2006 [25], incorporates triglycerides, BMI, GGT and waist circumference into a 0–100 score, with values ≥ 60 indicating a high likelihood of hepatic steatosis [28]. Although initially conceived for hepatologic screening, subsequent studies have demonstrated its broader relevance as a vascular-risk tool. For example, in hypertensive cohorts, higher FLI values independently predicted incident major cardiovascular events over approximately five years [29], and related evidence has linked FLI to subclinical target-organ damage, suggesting a shared mechanistic pathway involving metabolic dysfunction, neuro-humoral activation and vascular remodelling [30]. Our findings are consistent with these observations and may extend them to penile hemodynamic. Ates et al. reported a positive correlation between FLI and ED severity [16], while National Health and Nutrition Examination Survey (NHANES) data showed a dose–response relationship between steatosis burden assessed via U.S. Fatty Liver Index and ED prevalence [17]. Importantly, these previous investigations did not incorporate objective vascular diagnostics; thus, our study first suggests a mechanistic bridge between metabolic liver disease and VED.

The association between higher FLI values and reduced PSV is biologically plausible. FLI reflects insulin resistance, visceral adiposity and low-grade systemic inflammation—processes known to impair endothelial function through diminished nitric oxide (NO) bioavailability and increased oxidative stress, two central drivers of vasculogenic ED [31]. Hasanain et al. showed that insulin resistance independently predicts ED in patients with non-alcoholic fatty liver disease [32]. Russo et al. similarly reported insulin resistance as a predictor of ED in men with benign prostatic hyperplasia [33].

Potential mechanistic pathways include enhanced vascular expression of endothelin-β receptors and increased generation of reactive oxygen species, which together promote vasoconstriction and impair endothelial homeostasis. Animal models support this interpretation: obese insulin-resistant Zucker rats exhibit disrupted endothelin-1–mediated calcium signalling and exaggerated smooth-muscle contraction in penile arteries [34, 35]. These converging data suggest that hepatic steatosis and metabolic dysfunction may directly contribute to penile arterial insufficiency.

The clinical characteristics of men with pathological PSV in our cohort provide further support for this link. Although all subjects presented with impaired IIEF-5 scores, the subgroup with reduced PSV was older and exhibited a higher prevalence of hypertension and dyslipidaemia, consistent with the typical clinical profile of vasculogenic ED. The comparable distribution of overt diabetes between groups likely reflects limited sample size, whereas the higher fasting glucose values observed among men with pathological PSV suggest early metabolic impairment with vascular consequences.

Regarding IMT, patients with pathological values were older and had higher FSH concentrations, while steatosis indices remained comparable between groups. These findings align with emerging evidence suggesting that FSH exerts direct actions on the vascular endothelium. Preclinical studies by Rocca et al. demonstrated that FSH receptors are expressed in endothelial cells and that elevated FSH levels impair endothelial barrier integrity, alter vascular endothelial cadherin distribution, promote cytoskeletal remodelling and induce dysregulated NO production [36]. Clinical data summarized by Spaziani et al. further support an association between elevated FSH and adverse cardiometabolic profiles, including increased inflammation, lipid remodelling and atherosclerotic burden [37]. However, no significant correlation was observed between FSH and IMT as a continuous variable. This pattern is consistent with a group-level association rather than a linear relationship across the full IMT spectrum, possibly reflecting indirect or threshold-mediated vascular effects. Consistently, in a multivariable model including both age and FSH, neither variable remained independently associated with IMT, suggesting that the observed group-level association may reflect age-related endocrine changes rather than a direct vascular effect of FSH.

Beyond these primary observations, several additional findings strengthen the interpretation of FLI as a marker of penile vascular impairment. Men with reduced PSV displayed a markedly adverse metabolic and hormonal phenotype, characterized by significantly higher FSH and LH levels, lower total and free testosterone concentrations, and a higher burden of cardiometabolic abnormalities. Importantly, among the steatosis indices evaluated, FLI was the only marker that differed significantly between PSV groups, whereas HSI and DSI were comparable. This suggests that FLI captures a component of metabolic–vascular dysfunction not reflected by other indices; however, an alternative and non-mutually exclusive interpretation is that FLI more strongly reflects overall adiposity and lipid burden, which are themselves closely linked to vascular impairment. Although increased IMT was highly prevalent (77%), differences of IMT were primarily age-driven; however, the association between pathological IMT and higher FSH levels is noteworthy and suggests possible endocrine–vascular interactions. Finally, in multivariable analysis FLI remained the only independent predictor of PSV, supporting the association between metabolic steatosis indices and penile vascular dysfunction. The ROC-derived cut-off (FLI ≥ 67.6), with a specificity of 82%, further highlights the potential utility of FLI as a triage tool to identify men who may benefit from vascular assessment.

The present findings carry relevant clinical implications. FLI is available at no additional cost, derives from routinely collected clinical variables and may help identify men at increased risk of VED at an early stage. In settings where PCDU cannot be performed—such as in patients receiving anticoagulant therapy—an elevated FLI may help orient the diagnostic suspicion toward a vascular aetiology. However, given the modest discriminative performance observed, FLI should not be regarded as a diagnostic or screening tool and its potential clinical role remains exploratory.

Moreover, previous studies have shown that higher FLI values correlate not only with greater ED severity but also with lower circulating testosterone levels, suggesting its usefulness as an indirect marker of functional hypogonadism [20]. Integrating FLI into the evaluation of ED may therefore provide a more comprehensive assessment of metabolic and endocrine contributors to sexual dysfunction.

Several limitations must be acknowledged. The retrospective, single-centre design may restrict generalizability of the findings, and the relatively small sample size—particularly the limited number of participants with reduced PSV—may have reduced statistical power and affected the stability of multivariable models, especially for secondary endpoints such as IMT-related analyses. Accordingly, effect estimates and confidence intervals should be interpreted with caution. Moreover, although multivariable adjustment was performed, residual confounding inherent to observational research cannot be excluded. In addition, sensitivity analyses including BMI and waist circumference in the multivariable models attenuated the association between FLI and PSV. This result likely reflects the intrinsic overlap between adiposity measures and the components of the FLI formula, and the potential collinearity introduced when these variables are included simultaneously in the same model. Nevertheless, the use of standardized PCDU assessments, objective vascular endpoints, and comprehensive multivariable adjustment in a well-characterized and clinically homogenous cohort supports the internal validity and consistency of the observed associations, despite the inherent limitations of the study design. Larger prospective multicentre studies are needed to validate these findings and to clarify whether FLI may have a role within diagnostic or risk stratification frameworks for ED.

Until such evidence becomes available, the observed association between FLI and impaired penile vascular parameters should be regarded as exploratory or hypothesis-generating, and does not support the routine clinical use of FLI for the assessment or management of vasculogenic erectile dysfunction.

## Conclusion

FLI emerges as a practical, non-invasive and clinically meaningful marker associated with penile vascular parameters, suggesting a potential link with vascular andrological risk; however, its clinical utility remains unproven and requires confirmation in prospective studies. By capturing the metabolic and vascular dimensions of ED, FLI may enhance early identification of vasculogenic ED and serve as a bridge between hepatology, cardiology, endocrinology and andrology.

## Supplementary Information

Below is the link to the electronic supplementary material.

Supplementary Material 1

## Data Availability

Original data generated and analysed during this study are included in this published article.
