# Supplementary material for: Steatosis indices and penile vascular health: a metabolic association linking liver and vascular phenotypes
Source: J Endocrinol Invest. 2026 Apr 10;49(9):2385–95. doi: 10.1007/s40618-026-02889-1 (PMC13498629; doi:10.1007/s40618-026-02889-1)
Supplement: Supplementary file 1 [file 40618_2026_2889_MOESM1_ESM.docx]

**Supplementary Table S1**. Sensitivity analyses including BMI or waist circumference in the multivariable model for PSV

| **Variable** | **Model 1**  **Main model** | **Model 2**  **+ BMI** | **Model 3**  **+ Waist circumference** |
| --- | --- | --- | --- |
| FLI | β = −0.287; p = 0.031 | β = −0.384; p = 0.077 | β = −0.234; p = 0.288 |
| BMI | – | β = 0.124; p = 0.561 | – |
| Waist circumference | – | – | β = −0.063; p = 0.772 |
| Age | ns | ns | ns |
| Diastolic BP | ns | ns | ns |
| LH | ns | ns | ns |
| Total testosterone | ns | ns | ns |
| Free testosterone | ns | ns | ns |

FLI: fatty liver index, BMI: body mass index, BP: blood pressure; LH: Luteinizing Hormone
